# Supplementary material for: Examination of the cognitive function of Japanese community-dwelling older adults in a class for preventing cognitive decline during the COVID-19 pandemic
Source: PLoS One. 2021 Dec 13;16(12):e0248446. doi: 10.1371/journal.pone.0248446 (PMC8668118; doi:10.1371/journal.pone.0248446)
Supplement: Attachment — Submitted filename: Rubuttal_letter.docx [file pone.0248446.s003.docx]

September 9, 2021

Dear Editors and Reviewers,

Subject: Submission of revised paper ID: PONE-D-21-06873.

Please find attached a revised version of the article entitled “Examination of the cognitive function of Japanese community-dwelling older adults in a class for preventing cognitive decline during the COVID-19 pandemic,” which we would like to resubmit for publication in *PLOS ONE*.

We would like to thank the Reviewer/Editor for providing their comments. These comments enabled us to greatly improve the quality of this article. Please find enclosed our point-by-point responses to each of the comments provided by the Reviewers/Editor.

Revisions in the text are shown in red text for additions and in strikethrough for deletions. We hope that the revisions in the manuscript and the accompanying responses are sufficient to make this article suitable for publication in *PLOS ONE*.

We look forward to hearing from you.

Sincerely,

Minoru Kouzuki,

Department of Biological Regulation, School of Health Sciences, Faculty of Medicine, Tottori University, Tottori, Japan.

Email: kouzuki@tottori-u.ac.jp

**Responses to the Journal Requirements**

1. Please ensure that your manuscript meets PLOS ONE's style requirements, including those for file naming.

Response: Thank you for this reminder. We have checked PLOS ONE's style requirements. We made some corrections in the arrangement of contents within a cell in Tables 1, 2, and S2.

2. For this observational study, please avoid causal-sounding language (such as 'impact' or 'effect') when reporting associations, including in the title.

Response: As you suggested, we have revised the title and some parts of the text as much as possible.

3. Thank you for stating the following in the Competing Interests/Financial Disclosure* (delete as necessary) section:

“I have read the journal's policy and the authors of this manuscript have the following competing interests: MK, SF, and KM have no conflicts of interest to declare. KU owns a patent on the Touch Panel-type Dementia Assessment Scale and receives royalties from Nihon Kohden Corporation (Tokyo, Japan).”

We note that one or more of the authors are employed by a commercial company: Touch Panel-type Dementia Assessment Scale & Nihon Kohden Corporation (Tokyo, Japan)

a. Please provide an amended Funding Statement declaring this commercial affiliation, as well as a statement regarding the Role of Funders in your study. If the funding organization did not play a role in the study design, data collection and analysis, decision to publish, or preparation of the manuscript and only provided financial support in the form of authors' salaries and/or research materials, please review your statements relating to the author contributions, and ensure you have specifically and accurately indicated the role(s) that these authors had in your study. You can update author roles in the Author Contributions section of the online submission form.

Please also include the following statement within your amended Funding Statement.

“The funder provided support in the form of salaries for authors [insert relevant initials], but did not have any additional role in the study design, data collection and analysis, decision to publish, or preparation of the manuscript. The specific roles of these authors are articulated in the ‘author contributions’ section.”

If your commercial affiliation did play a role in your study, please state and explain this role within your updated Funding Statement.

Response: The Touch Panel-type Dementia Assessment Scale (TDAS) is the name of the cognitive function test, and Nihon Kohden Corporation is the company that sells TDAS. KU receives royalties from Nihon Kohden Corporation, but is not employed. In addition, this study did not use any royalties and was not funded by Nihon Kohden Corporation. This study was independently planned and carried out by researchers who were not employed by a commercial company, and Nihon Kohden Corporation had no role in the study design, data collection and analysis, decision to publish, or preparation of the manuscript. Therefore, there are no changes to the Funding Statement as the following: “The authors received no specific funding for this work.”

b. Please also provide an updated Competing Interests Statement declaring this commercial affiliation along with any other relevant declarations relating to employment, consultancy, patents, products in development, or marketed products, etc.

Within your Competing Interests Statement, please confirm that this commercial affiliation does not alter your adherence to all PLOS ONE policies on sharing data and materials by including the following statement: "This does not alter our adherence to PLOS ONE policies on sharing data and materials.” (as detailed online in our guide for authors http://journals.plos.org/plosone/s/competing-interests). If this adherence statement is not accurate and there are restrictions on sharing of data and/or materials, please state these. Please note that we cannot proceed with consideration of your article until this information has been declared.

Please include both an updated Funding Statement and Competing Interests Statement in your cover letter. We will change the online submission form on your behalf.

Response: Within our Competing Interests Statement, we added, "This does not alter our adherence to PLOS ONE policies on sharing data and materials.” Please refer to the following:

“MK, SF, and KM have no conflicts of interest to declare. KU owns a patent on the Touch Panel-type Dementia Assessment Scale and receives royalties from the Nihon Kohden Corporation (Tokyo, Japan). This does not alter our adherence to PLOS ONE policies on sharing data and materials.”

4. Please review your reference list to ensure that it is complete and correct. If you have cited papers that have been retracted, please include the rationale for doing so in the manuscript text, or remove these references and replace them with relevant current references. Any changes to the reference list should be mentioned in the rebuttal letter that accompanies your revised manuscript. If you need to cite a retracted article, indicate the article’s retracted status in the References list and also include a citation and full reference for the retraction notice.

Response: We checked the reference list and no retracted articles were included. However, some errors in the reference list were revised.

**Responses to the comments of Reviewer 1**

1. The opening statement of the manuscript, the first line of the manuscript reads incorrect. Use of too many 'ands' in the statement. Also, what is 'thoughts on cognitive function'? Kindly modify the statement, it reads very vague.

Response: We apologize for the lack of clarity. We have revised the text in the opening part of the Abstract.

2. Authors have not mentioned age of the individuals in the abstract and age and number of patients recruited in the MM section. They only mention it finally in the result section. Kindly add this information in other two sections.

Response: We appreciate this valuable suggestion. We have added information on the age of the individuals in the Abstract, as well as the age and number of participants recruited in the Methods/Study participants section. In addition, we have revised the Results/Participant characteristics section to avoid duplication of text.

3. Format of Table-1 can be improved. Kindly make it concise.

Response: Thank you for your comment. We have tried to make the format of Table 1 as concise as possible. In addition, we have changed the sex description format in Table 2.

**Responses to the comments of Reviewer 2**

1. This title seems better "Impact of the COVID19 pandemic on cognitive function of Japanese community-dwelling older adults in a class for preventing cognitive decline".

Response: Thank you for your comment. However, the Journal Requirements pointed out to avoid causal-sounding language (such as 'impact' or 'effect') when reporting associations. Therefore, we considered the following title and short title as alternatives:

Title: Examination of the cognitive function of Japanese community-dwelling older adults in a class for preventing cognitive decline during the COVID-19 pandemic

Short title: Examination of cognitive function of Japanese older adults during the COVID-19 pandemic

2. In lines 43-44, “Participation in the class leads to the formation of a community”. It would be helpful to specify or briefly mention/explain the class structure. Also in line 52> specify or briefly mention/explain the class structure.

Response: We appreciate this valuable suggestion. We have changed the text related to the class to improve its coherence. Specifically, we added a description of the class structure on line 38 and corrected the text on lines 44 and 54.

3. At many places In the intro and other sections “the class(es)” can be changed to ‘this(these) class(es)’’ after having briefly mentioned what those classes are at the beginning.

Response: We agree with your recommendation. We already explained the class at the beginning of the introduction and Methods/Study participants; thus, we changed some from “the class(es)” to “this(these) class(es)” as advised.

4. In line 86, delete “not”.

Response: Thank you for this comment. We have revised the sentences accordingly in line 89.

5. I didn’t not understand this line (112-113) “The exclusion criteria were subjects who declined the use of their data”.

Response: We apologize for the lack of clarity. In this study, all information pertaining to the study was disclosed in advance on the homepage of the Tottori University website and the notice board of the Houki town hall to ensure that participants were given the opportunity to decline participation (opt-out approach). Therefore, the exclusion criteria meant that participants who declined the use of their data were excluded. This was the item that we described in the application form for ethics review (the research proposal); thus, we have included it in the text as well. We have added an explanation of the exclusion criteria to improve its coherence.

6. From Table 1 & 2, I couldn’t get the clear idea as to what the observed cognitive parameters were before and/or after the class suspension.

Response: As we wrote in the Methods section, cognitive function was evaluated using only TDAS. However, we found the description difficult to understand; thus, we have revised the text of the Methods/Data Collection and added annotations for the decline and non-decline groups in Table 2.

7. I am also concerned about the small sample size of cognitive decline group. I was also wondering if three months (early March to end May) is good enough time window to assess the dementia related symptoms.

Response: Thank you for this very important comment. As the reviewer has pointed out, we also considered the small sample size of the cognitive decline group as a limitation of our study (described as the third limitation in the Discussion section). Unfortunately, we were unable to collect any additional data. We have added a little description about the sample size; thus, the reader should pay attention to the interpretation of the results. Regarding the time window, three months may be a short period, but because daily life has changed significantly due to COVID-19, we performed this study because we believe that even class suspension of three months would have a negative effect on cognitive function. In fact, a study has been reported that in a study investigating the effects of about two months of COVID-19 lockdown in Italy, telephone interviews with caregivers of 31 subjects with MCI revealed a worsening of cognition in 41.9% of individuals (ref 30: Baschi R, et al. Front Psychiatry. 2020; 11: 590134.).

**Corrections other than reviewer comments**

Some errors in Table 2, abbreviation notation (lines 48 and 125), typographical errors (lines 156 and 287), and reference lists (lines 399, 404, 410, 428, 441, 443, 463, 467, and 511) have been corrected. However, these changes did not affect the interpretation of the results. We apologize for the lack of confirmation.
