# Supplementary material for: Examination of the cognitive function of Japanese community-dwelling older adults in a class for preventing cognitive decline during the COVID-19 pandemic
Source: PLoS One. 2021 Dec 13;16(12):e0248446. doi: 10.1371/journal.pone.0248446 (PMC8668118; doi:10.1371/journal.pone.0248446)
Supplement: Attachment — Submitted filename: Rubuttal_letter2.docx [file pone.0248446.s004.docx]

October 7, 2021

Dear Editors and Reviewers,

Subject: Submission of revised paper ID: PONE-D-21-06873R1.

Please find attached a revised version of the article entitled “Examination of the cognitive function of Japanese community-dwelling older adults in a class for preventing cognitive decline during the COVID-19 pandemic,” which we would like to resubmit for publication in *PLOS ONE*.

We would like to thank the Reviewer/Editor for providing their comments. These comments enabled us to greatly improve the quality of this article. Please find enclosed our point-by-point responses to each of the comments provided by the Reviewers/Editor.

Revisions in the text are shown in red text for additions and in strikethrough for deletions. We hope that the revisions in the manuscript and the accompanying responses are sufficient to make this article suitable for publication in *PLOS ONE*.

We look forward to hearing from you.

Sincerely,

Minoru Kouzuki,

Department of Biological Regulation, School of Health Sciences, Faculty of Medicine, Tottori University, Tottori, Japan.

Email: kouzuki@tottori-u.ac.jp

**Responses to the Journal Requirements**

1. Please review your reference list to ensure that it is complete and correct. If you have cited papers that have been retracted, please include the rationale for doing so in the manuscript text, or remove these references and replace them with relevant current references. Any changes to the reference list should be mentioned in the rebuttal letter that accompanies your revised manuscript. If you need to cite a retracted article, indicate the article’s retracted status in the References list and also include a citation and full reference for the retraction notice.

Response: We checked the reference list, and no retracted articles were included. However, the details of reference 40 were revised.

**Responses to the comments of Reviewer 2**

In the revised manuscript “By participating in this class…” in line 44 now becomes incoherent. It has to be preceded with a sentence explaining what ‘this class’ actually is. Now that the authors have changed the structure of this sentence-in the process made it more complex ‘this’ in the sentence may be changed to ‘the’ OR better simplify this sentence.

Response: Thank you for your comment. We have revised the sentences in lines 44-47.

In my previous review, I had asked the authors to change ‘the class(es) to this (these) class(es) at certain places giving readers a clear reference as to what those classes are, which the authors have actually done. In the revised manuscript, however, I noticed that the authors have made these changes at many other places where it was not required at all (e.g in line 65). The authors must fix such inconsistencies before the manuscript can be accepted for publication.

Response: We apologize because we did not understand your intentions correctly. We changed some from “this class” to “the class” as you have pointed out. If the correction is incorrect, please let me know in detail.

Lines 116-118 are contradictory.

Response: We completely agree with you. We have revised the sentences in lines 116-118.
